# Evaluating the Effectiveness of a Hybrid Tai Chi Cardiac Rehabilitation Programme for Psychological Stress Reduction and Oxidative Stress in Patients With Chronic Coronary Syndrome: A Randomized Controlled Trial

**DOI:** 10.1002/smi.70088

**Published:** 2025-09-08

**Authors:** Meize Cui, Cuihan Li, Yameng Li, Zaihao Chen, Qiuyang Wei, Mingyu Liu, Hui Fang, Lujia Li, Yuerong Huang, Shaojun Lyu, Jianwei Zhang

**Affiliations:** ^1^ College of Physical Education and Sports Beijing Normal University Beijing China; ^2^ Police Sports Teaching and Research Department The National Police University for Criminal Justice Baoding China; ^3^ Department of Physical Education Northwestern Polytechnical University Xi'an China; ^4^ Sports Department Jiangsu University Zhenjiang China

**Keywords:** cardiac rehabilitation, chronic coronary syndrome, oxidative stress, perceived stress, tai chi

## Abstract

Preliminary evidence suggests that Tai Chi may effectively relieve pain symptoms, increase quality of life, and reduce cardiovascular risk in patients with chronic coronary syndrome (CCS). However, few randomized controlled trials have specifically investigated the potential benefits of Tai Chi in patients with CCS, particularly regarding its effects on psychological stress and cellular stress levels. To evaluate the effectiveness of a hybrid Tai Chi cardiac rehabilitation programme in reducing perceived stress and oxidative stress in diagnosed patients. Forty‐six patients with CCS were randomly assigned to 12 weeks of either a Tai Chi cardiac rehabilitation programme (TCCRP, *n* = 23) or a conventional exercise cardiac rehabilitation programme (CECRP, *n* = 23) (3 sessions per week). All participants continued their routine drug treatments daily. The main outcome measure was the Chinese Perceived Stress Scale (CPSS). The secondary outcome measures included the antioxidant enzymes catalase (CAT) and glutathione peroxidase (GSH‐Px) and the stress marker oxidised low‐density lipoprotein (ox‐LDL). The data were analysed by 2‐way mixed analysis of variance with post hoc Bonferroni adjustment and paired *t* tests. The group‐by‐time interaction effect on CPSS was significantly different (MD = −7.71, 95% CI [−10.750, −4.678], *p* < 0.001). Within the TCCRP group, the CPSS score significantly decreased (*p* < 0.05) from baseline to the end of the intervention. Notably, in the CECRP group, the CPSS score increased (*p* < 0.05) at the end of the intervention. The CAT and GSH‐Px levels increased markedly in the TCCRP group after the intervention (*p* < 0.001). Spearman's correlation analysis revealed that CPSS was positively correlated with ox‐LDL (*r* = 0.569, *p* < 0.05) and negatively correlated with GSH‐Px (*r* = −0.585, *p* < 0.05). The correlation in the control group was not statistically significant (*r* = −0.148, *p* > 0.05). A 12‐week hybrid Tai Chi cardiac rehabilitation programme can effectively regulate psychological stress perception and reduce physiological stress levels in patients with coronary heart disease.

## 1 Introduction

Epidemiology has shown that stress significantly influences the occurrence, progression, and prognosis of coronary heart disease (Vaccarino et al. 2021; Wirtz and Von Känel 2017). The 2019 guidelines from the European Society of Cardiology (ESC) redefined stable coronary heart disease as chronic coronary syndrome (CCS) (Okutucu and Görenek 2019). In addition to antianginal, lipid‐lowering, and antithrombotic therapies, the current CCS guidelines emphasise the importance of prevention through lifestyle modifications (Taqueti and Ridker 2017). Furthermore, recent research highlights that traditional cardiovascular risk factors, such as age, diabetes, hypertension, hyperlipidaemia, and smoking, are compounded by the finding that high‐stress perception is an independent risk factor for acute myocardial infarction, particularly among young Chinese men (National Centre for Cardiovascular Diseases [NCCD], 2024; Almuwaqqat et al. 2020). Numerous studies investigating the pathogenesis of coronary heart disease have identified microvascular pathological features associated with elevated levels of oxygen free radicals (Afanas’ev 2011). Under normal circumstances, the body's antioxidant system effectively eliminates the most harmful reactive oxygen species (ROS). However, under pathological conditions, endothelial dysfunction disrupts vascular elasticity, resulting in oxidative stress, the activation of inflammatory mediators, and the formation of vascular blockages (Deng et al. 2016; Clemente‐Suárez et al. 2023; Begum et al. 2022).

Research indicates that the regulation of reactive oxygen species (ROS) in response to exercise is influenced by several factors, including the type of exercise, exercise intensity, total duration, and individual fitness level (Franklin et al. 2020). Specifically, engaging in a single session of high‐intensity exercise, performing targeted local resistance exercises on specific muscle groups, or exceeding certain thresholds of intensity or duration can exacerbate oxidative stress and muscle damage (Roh et al. 2017; Diaba‐Nuhoho et al. 2018). Conversely, regular aerobic exercise at moderate intensity, along with various forms of resistance training, has been shown to enhance the antioxidant defence system, foster tolerance to oxidative damage, and promote adaptive physiological responses to combat oxidative stress (Boccatonda et al. 2016; Masuda et al. 2006). An imbalance between the oxidation and antioxidant systems leads to increased tissue damage (Huang and Nan 2019; Kibel et al. 2020). Importantly, vascular walls contain various enzymes, such as super catalase (CAT) and glutathione peroxidase (GSH‐Px), which help reduce the burden of ROS and contribute to the body's antioxidant defence system (Dinarvand et al. 2021). However, oxidised low‐density lipoprotein (ox‐LDL) is cytotoxic to endothelial cells; oxidative stress can lead to the oxidation of low‐density lipoprotein (LDL). The oxidation of LDL attracts monocytes, causing the accumulation of inflammatory cells in blood vessels (S. Gao and Liu 2017).

Previous studies have shown that exercise rehabilitation improves the prognosis of patients with CCS (Sandesara et al. 2015). Alongside current exercise programs, Tai Chi can serve as an additional option for patients at low to moderate risk. Moreover, transcendental meditation might be employed as a technique for reducing stress (Arthur et al. 2006). This study highlights the safety and effectiveness of a Tai Chi‐based cardiac rehabilitation programme for patients with chronic coronary syndrome and is a continuation of a previously published study (J. Ma et al. 2020). However, it remains unclear whether the TCCRP can reduce stress perception, increase antioxidant enzyme activity, and decrease lipid damage in patients with CCS. Additionally, there is a lack of clinical empirical research comparing its effects to those of conventional exercise rehabilitation.

Thus, this study aimed to investigate the impact of different forms of exercise intervention, including a 12‐week hybrid exercise rehabilitation programme, on the Chinese Perceived Stress Scale (CPSS) score and oxidative stress in patients with CCS. The goal of this study was to evaluate the effectiveness of the Tai Chi rehabilitation programme on the physical and mental health of patients with CCS.

## 2 Methods

The study is a hybrid Tai Chi Cardiac Rehabilitation Programme for reducing psychological stress and oxidative stress in patients with CCS, follows the Helsinki Declaration and has been approved by the Ethics Committee of the Chinese People's Liberation Army General Hospital (approval number: S2019‐060‐02), with registration at ClinicalTrials.gov (NCT Number: NCT03936504) (Submitted: 2019‐05‐01) prior to enrolling the first participant. All participants received comprehensive information regarding the exercise intervention and provided their informed consent by signing the appropriate documents.

### 2.1 Study Design

**FIGURE 1:**
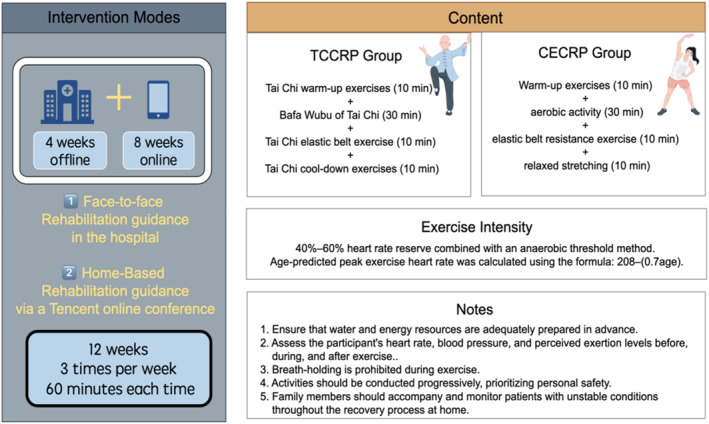
Intervention plan.

This randomized, controlled, 2‐site, parallel‐group trial of exercise rehabilitation along with daily drug intervention was conducted among 46 patients with CCS enroled from 2 community hospitals in China. The participants were randomly assigned to two groups (1:1 ratio): an experimental group (Tai Chi Cardiac Rehabilitation Programme, TCCRP) and a control group (Conventional Exercise Cardiac Rehabilitation Programme, CECRP). The intervention lasted for 12 weeks. The participants were allocated using the simple randomisation method. The specific exercise intervention plan is shown in Figure 1 (By Figdraw).

### 2.2 Participants

From October 2020 to November 2021, a total of 46 patients with CHD were enroled from two community hospitals in Beijing and Shandong, China. The diagnostic criteria for chronic coronary syndrome were based on the ‘2019 ESC Guidelines for the Diagnosis and Management of Chronic Coronary Syndrome (CCS)’ issued by the European Society of Cardiology (ESC) in 2019 (Möllmann et al. 2020). The inclusion criteria included male or nonpregnant female participants aged between 30 and 80 years with NYHA classes I and II (Cui et al. 2024). Participants were excluded if they had acute myocardial infarction, severe aortic stenosis, hypertrophic cardiomyopathy, severe valvular heart disease, malignant tachyarrhythmia, abnormal motor function caused by neurological deterioration, motor system disease, or rheumatism within 2 weeks. Participants were also excluded if they had any medication changes in the previous 3 months, attended psychological or physical therapy, had a history of steady exercise, or had received exercise training in the last 3 months. All patients who met the inclusion and exclusion criteria and provided written informed consent were included in the study.

**FIGURE 2:**
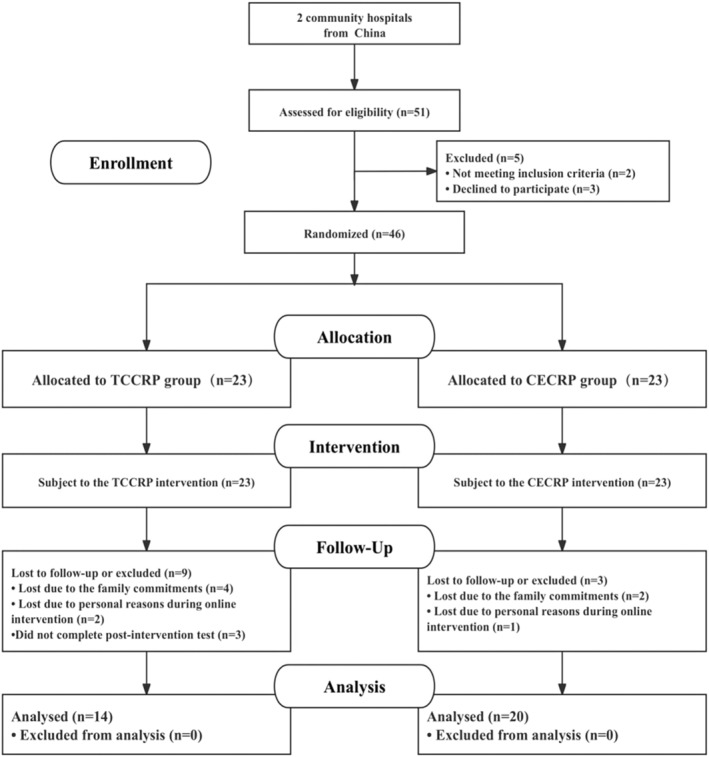
Flow of participants through the study. CECRP: Conventional exercise cardiac rehabilitation programme. TCCRP: Tai Chi cardiac rehabilitation programme. The reasons for not completing the postintervention test (*n* = 3) were as follows: not able to travel to the test point on time (*n* = 2); and did not provide a reason (*n* = 1).

The flow of participants throughout the study is presented in Figure 2. Out of the initial 46 patients, 34 patients completed 12‐week interventions with pre‐ and postintervention assessments, which included 14 patients in the TCCRP group and 20 patients in the CECRP group. The research process included recruitment, screening, randomisation, intervention, and follow‐up. Prior to each exercise intervention, therapists conducted regular lifestyle habit questionnaires and assessments of the patients, which included dietary patterns, medications, smoking, and alcohol consumption. The participants in both groups were instructed not to alter their regular lifestyle habits during the study.

### 2.3 Sample Size Calculation

The sample size was calculated based on the changes in CPSS (Mean ± SD) between the comparison groups. According to published literature (Cheung et al. 2019), the mean CPSS scores for the control and intervention groups post‐intervention were (18.62 ± 1.04, 17.59 ± 1.02), respectively. Using G*Power analysis (Faul et al. 2009), it was determined that 34 subjects were required to achieve a power of 80% at a significance level of 5%. Considering an anticipated follow‐up loss rate of approximately 30%, the sample size was increased to 46, resulting in 23 participants being allocated to each group.

### 2.4 Randomisation

The random allocation sequence was generated by an independent statistician using the PLAN sentences of the statistical software SAS V.9.2. The allocation assignments will then be sent to a study staff member, who will store them in sealed envelopes. These envelopes will have date and signature labels placed over the seals. The randomisation envelopes will only be opened if a participant meets the eligibility criteria, completes the informed consent, and undergoes a baseline assessment. Laboratory personnel were blinded to the group assignments.

### 2.5 Intervention

The participants were randomized into 2 groups. The TCCRP group practiced the planned Tai Chi exercise module for 60 min, 3 times a week, under the supervision of a trained Tai Chi instructor. The CECRP group practiced the planned aerobic exercise module for 60 min, 3 times a week, under the supervision of a trained PE doctor instructor.

#### 2.5.1 Intervention Modes

The intervention lasted for a total of 12 weeks, with 4 weeks of hospital rehabilitation guidance followed by 8 weeks of home‐based remote online real‐time rehabilitation guidance (Cui et al. 2024). Prior to the intervention, patients underwent a cardiopulmonary exercise test (CPET) to assess their exercise tolerance. Based on the results of the CPET, as well as echocardiography results, previous medical history, and medication history of both patient groups, exercise prescriptions were developed (Zhang et al. 2021).

#### 2.5.2 Exercise Modules

Two different exercise modules were developed for the two intervention programs. Each training session lasted 60 min and included ordinary warm‐up exercises (10 min), aerobic activity (30 min), resistive exercise (10 min), and cool‐down exercises (10 min). The TCCRP consisted of (a) traditional Tai Chi warm‐up exercises, followed by (b) Bafa Wubu of Tai Chi (Lyu et al. 2020): It emphasises the integration of the body, breath, and mind. This is particularly important as Bafa Wubu requires individuals to ‘cultivate their body’, ‘regulate their breath’, and ‘engage their mind’ (Lyu 2018), (c) Tai Chi elastic belt exercises, and (d) Tai Chi cool‐down exercises.

The CECRP included (1) an active warm‐up that involves arm swinging and gentle stretches of the neck, shoulders, spine, arms, and legs; (2) an aerobic activity focused primarily on aerobics, such as side‐to‐side steps, clapping under the thighs, lateral jumping jacks, back kicks, and vertical jumping jacks. These exercises emphasise coordinated movements, a consistent rhythm, and smooth breathing, while avoiding breath‐holding during physical activity. Progress should be gradual during practice, ensuring accurate range of motion and stability in the transfer of the centre of gravity (Franklin et al. 2022); (3) resistive exercise consisting of resistance band exercises; and (4) a cool‐down session that included active and static stretching exercises with primary body movements.

### 2.6 Safety Supervision and Precautions

During the exercise intervention, accompanying staff and medical personnel monitored changes in blood pressure, blood oxygen, and heart rate before, during, and after each exercise session. The patients' subjective feelings during and after exercise were evaluated using the rated perceived exertion (RPE) scale.

Throughout the entire experimental period, the patient's physical activity level and any adverse events were recorded and monitored using the rehabilitation record sheet. Each patient's physical activity level was categorised as less than 3 times per week, 3 times per week to 5 times per week, and greater than 5 times per week. Notably, patients with unstable conditions were required to be accompanied and monitored by their family members during the recovery process at home.

### 2.7 Outcome Measures

#### 2.7.1 CPSS

Patients were assessed using the Chinese Perceived Stress Scale (CPSS), which was originally developed by Cohen in 1983 and later revised by Yang Tingzhong. This scale is widely recognized and extensively used internationally for measuring individual stress levels. The reliability of the CPSS was tested for reliability and had an intraclass correlation coefficient (ICC) of 0.78 (Y. Gao et al. 2022; Cohen et al. 1983). The scale consists of 14 items that are divided into two categories: sense of tension and loss of control. Higher scores indicate higher levels of stress. The CPSS was evaluated at baseline, 4 and 12 weeks (at the end of the intervention).

#### 2.7.2 Blood Sample Collection and Oxidative Stress Evaluation

Observation indicators, including CAT, GSH‐Px, and ox‐LDL, were measured in this study. Peripheral blood samples were collected from the subjects in the early morning after an overnight fast, both 1 week before and 1 week after exercise rehabilitation. A total of 5 mL of blood was drawn using a separation gel anticoagulation tube. The sample was allowed to stand for 1–2 h to allow for coagulation and stratification. The sample was subsequently centrifuged at a low temperature (3000 rpm) for 10 min. The serum was collected after separation and aliquoted into EP tubes for testing. Quantitative detection of the observation indicators was performed using the enzyme‐linked immunosorbent assay (ELISA) method. The ELISA kits used in this study were provided by Beijing Huaying Biotechnology Research. All the observation indicators were measured at baseline and at 12 weeks.

### 2.8 Statistical Methods

Analysis was performed using CPSS 29.0 (IBM CPSS Statistics for Windows, USA), with a statistical significance level set at *p* < 0.05. Continuous variables are reported as the means and standard deviations, whereas medians were used for nonnormal distributions. Categorical variables are described as frequencies. All the data were checked for the presence of outliers, normal distributions (Kolmogorov–Smirnov test), and homogeneity of variance (Levene's test). Descriptive statistics were computed for all characteristic variables at baseline. Inter‐ and intragroup mean differences were analysed using Student's *t* test or analysis of variance (ANOVA), as appropriate. This study also conducted per‐protocol (PP) analysis to estimate the results of participants who did not comply with the protocol.

## 3 Results

### 3.1 Characteristics of the Study Participants

No significant differences were observed in age, sex, disease history, or other variables between the two groups (*p* > 0.05). Table 1 provides baseline values for these characteristics.

**TABLE 1:** Basic information on patients with CCS in the two groups.

Items	TCCRP group (*n* = 14)	CECRP group (*n* = 20)	*p value*
Sex			
Male, *n* (%)	12 (85.7)	14 (70.0)	0.422
Female, *n* (%)	2 (14.3)	6 (30.0)	
age	62.07 ± 9.076	61.90 ± 9.700	0.959
SBP (mmHg)	119.71 ± 16.582	120.50 ± 19.880	0.830
DBP (mmHg)	75.07 ± 9.401	76.35 ± 11.918	0.340
BMI (kg/m^2^)	26.65 ± 2.926	26.090 ± 2.524	0.555
LVEF (%)	62.778 ± 4.902	61.200 ± 5.970	0.948
Smoking history			
Never smoker, *n* (%)	6 (42.86)	10 (50.00)	0.738
Used to smoke, have now quit, *n* (%)	8 (57.14)	10 (50.00)	
Currently smoking, *n* (%)	0	0	
Drinking history			
Never drinker, *n* (%)	4 (28.57)	11 (55.00)	0.308
Used to drink, have now quit, *n* (%)	3 (21.43)	3 (15.00)	
Currently drinking, *n* (%)	7 (50.00)	6 (30.00)	
Type of revascularisation			
PCI, *n* (%)	7 (50.00)	7 (25.00)	0.487
CABG, *n* (%)	1 (7.14)	4 (20.00)	0.379
None, *n* (%)	6 (42.86)	9 (45)	0.738
Combined disease			
Hypertension, *n* (%)	10 (71.43)	14 (70.00)	1.000
Diabetes, *n* (%)	3 (21.43)	6 (30.00)	0.704
Hyperlipaemia, *n* (%)	9 (64.29)	15 (75.00)	0.704
None, *n* (%)	2 (14.29)	1 (5.00)	0.555
Combination medication			
Antiplatelet drugs, *n* (%)	13 (92.86)	15 (75.00)	0.364
Beta‐blockers, *n* (%)	9 (64.29)	13 (65.00)	1.000
Statins, *n* (%)	11 (78.57)	18 (90.00)	0.627
Nitrates, *n* (%)	5 (35.71)	10 (50.00)	0.495
ACEI/ARB, *n* (%)	4 (28.57)	5 (25.00)	1.000

Abbreviations: ACEI: angiotensin‐converting enzyme inhibitor; ARB: angiotensin II receptor blocker.BMI: body mass index; CABG: coronary artery bypass grafting; DBP: diastolic blood pressure; LVEF: left ventricular ejection fraction; PCI: percutaneous coronary intervention; SBP: systolic blood pressure.

### 3.2 Comparison of CPSS Scores Between the Two Groups

Comparisons of stress perception scores were conducted between the two groups of patients at baseline (T0), 4 weeks (T1), and 12 weeks (T2). Table 2 shows that there was no statistically significant difference in stress perception scores within each group before the intervention or at 4 weeks postintervention (*p* > 0.05). However, the TCCRP group presented a significant reduction in CPSS scores after 12 weeks (T0: 25.07 ± 7.85, T1: 26.86 ± 3.51, T2: 19.79 ± 3.93, *p* < 0.01), whereas the CECRP group presented a significant increase in CPSS scores at the same time point (T0: 24.55 ± 4.86, T1: 24.40 ± 4.78, T2: 27.50 ± 4.50, *p* < 0.05). The study utilised two‐factor repeated measures analysis of variance to assess the impact of exercise intervention programs on patients' stress perception scores. Data analysis confirmed a normal distribution, homogeneity of variance, and sphericity assumption. An interaction effect between group and time was observed (Ftime*group = 15.627, *p* < 0.001), leading to a separate effect analysis.

**TABLE 2:** Two‐way repeated‐measures ANOVA of CPSS scores (M ± SD).

Group	*n*	CPSS scores		
		T0	T1	T2
TCCRP group	14	25.07 ± 7.85	26.86 ± 3.51	19.7 ± 3.93**^ΔΔ###^
CECRP group	20	24.55 ± 4.86	24.40 ± 4.78	27.50 ± 4.50*^ΔΔ^
F	F_time*group_ = 15.627		F_time_ = 1.257	
P	P_time*group_ = 0.0003		P_time_ = 0.271	

^###^ *p* < 0.001 for comparisons between groups.

* *p* < 0.05 for comparisons between T2 and T0 within the group.

** *p* < 0.01 for comparisons between T2 and T0 within the group.

^Δ^ *p*< 0.05 for comparisons between T2 and T1 within the group.

^ΔΔ^ p< 0.01 for comparisons between T2 and T1 within the group.

**FIGURE 3:**
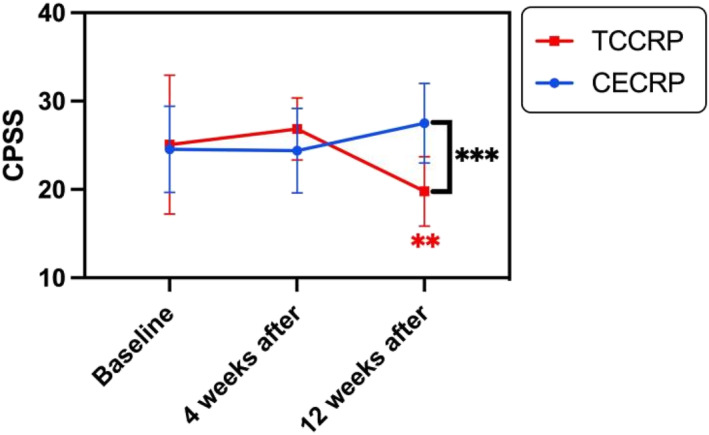
Repeated measurement analysis results of CPSS scores in the two groups at three time points. ***p < 0.001 for comparisons between groups; **p < 0.01 for comparisons between T2 and T0 within the TCCRP group.

The simple effect analysis of time indicated statistically significant differences in CPSS scores between the two groups at different time points (*p* < 0.01). Pairwise comparisons using the ‘LSD (Least Significant Difference)’ method revealed statistically significant differences in weight increase between the groups at different time points (*p* < 0.001). Figure 3 illustrates the separate effects of group and post hoc test analysis, which revealed no significant difference in CPSS scores between the two groups at baseline (*p* = 0.812). After 4 weeks, the scores remained nonsignificant (*p* = 0.112), but after 12 weeks, the TCCRP group had significantly lower CPSS scores than did the CECRP group by 7.71 (95% CI: −10.750 to −4.678), with a statistically significant difference (*p* < 0.001). Thus, the CECRP group demonstrated a greater reduction in patients' stress perception index.

### 3.3 Comparison of Oxidative Stress in the Two Groups

Prior to the exercise rehabilitation programme, there were no statistically significant differences in the serum levels of CAT, GSH‐Px, or ox‐LDL between the two groups (*p* > 0.05).

Following the 12‐week intervention, both groups presented significant increases in the serum CAT and GSH‐Px levels (*p* < 0.05). Although there was a decrease in the ox‐LDL concentration, the difference was not statistically significant (*p* > 0.05). Compared with the CECRP group, the TCCRP group presented a significantly greater change in the CAT concentration, with a statistically significant difference (*p* < 0.05). However, the differences in the changes in the GSH‐Px and ox‐LDL concentrations between the two groups were not statistically significant (*p* > 0.05), as shown in Table 3.

**TABLE 3:** Comparison of oxidative stress between the two groups (M ± SD).

Group	*n*	ΔCAT	ΔGSH‐Px	Δox‐LDL
TCCRP group	14	8.17 ± 5.45**^#^	45.05 ± 40.60**	−0.44 ± 0.28
CECRP group	20	3.97 ± 5.12**^#^	24.47 ± 41.43**	−0.14 ± 0.13
*t*		2.293	1.437	1.414
*p*		0.029	0.160	0.167

*Note:* *p < 0.05, **p < 0.01, after intervention versus before intervention; #*p* < 0.05, TCCRP versus CERP. CAT: catalase; GSH‐Px: glutathione peroxidase; ox‐LDL: oxidised low‐density lipoprotein.

### 3.4 Correlation Analyses Between Changes in the CPSS Score and Oxidative Stress After the Two Types of Exercise Intervention

We subsequently analysed the correlations between CPSS and oxidative stress indicators postintervention in the two groups. In the TCCRP group, a significant negative correlation was found between the CPSS score and the GSH‐Px score (*r* = −0.585, *p* = 0.028), indicating a statistically significant moderate correlation. The CPSS score was significantly positively correlated with ox‐LDL (*r* = 0.569, *p* = 0.034), indicating a statistically significant moderate correlation. The correlation coefficient between the CPSS score and CAT score was not statistically significant (*r* = −0.459, *p* = 0.099). Similarly, the correlation coefficients between various indicators in the CECRP group were not statistically significant (*p* > 0.05). These results are presented in Table 4.

**TABLE 4:** Correlations between changes in CPSS values and oxidative stress after the two types of exercise intervention.

Group	Indicators	ΔCAT		ΔGSH‐Px		Δox‐LDL	
		r	*p*	r	*p*	r	*p*
TCCRP group	ΔCPSS	−0.459	0.099	−0.585*	0.028	0.569*	0.034
CECRP group	ΔCPSS	−0.152	0.522	−0.017	0.942	−0.149	0.532

Abbreviations: CAT: catalase; GSH‐Px: glutathione peroxidase; ox‐LDL: oxidised low‐density lipoprotein.

* *p* < 0.05.

**p < 0.01.

## 4 Discussion

In this study, we investigated the effects of two exercise rehabilitation programs on psychological and physiological stress in patients with CCS using a randomized controlled trial design. Current exercise prescriptions for cardiac rehabilitation primarily consist of conventional Western rehabilitation programs that incorporate aerobic exercise, resistance training, stretching, and other modalities. The Tai Chi cardiac rehabilitation programme, which is specifically designed for patients with coronary heart disease, uniquely integrates Tai Chi, breathing exercises, and other traditional health‐preserving practices with Western elastic band resistance training techniques. The cardiac rehabilitation programme associated with Tai Chi highlights the necessity of fostering a tranquil mind and a relaxed body, removing any distractions, and concentrating on mental points to achieve optimal bodily alignment.

Stress is a significant factor in the development, progression, and prognosis of coronary heart disease (Ummer et al. 2021; Pasternac and Talajic 1991). Research has shown that individuals with coronary heart disease, particularly following percutaneous coronary intervention (PCI), tend to experience heightened stress levels compared with the general population (Pedersen et al. 2004). Failure to address this issue can have a profound effect on the mental well‐being of these patients (Packard et al. 2021). Numerous studies have demonstrated that addressing stress levels can alleviate psychological distress, enhance stress‐coping mechanisms, and ultimately lead to improved clinical outcomes and quality of life for patients (Pogosova et al. 2014; W. Zhang and H. Zhang 2022).

The findings of this research indicate that varying durations of exercise intervention programs are associated with changes in stress perception levels. Following a 1‐month in‐hospital rehabilitation period, there was no significant difference in stress perception between the two patient groups. However, after a 2‐month home rehabilitation period, the experimental group exhibited a notable reduction in stress perception compared with the control group. We tentatively infer that the more significant psychological regulation effects of Tai Chi may be related to breath and consciousness modulation (Qi et al. 2023; Yao et al. 2021), while the CECRP group may not have a notable impact on psychological regulation. These findings still require validation with larger sample sizes. Additionally, future studies should include a blank control group for more rigorous experimental design.

To delve deeper into the impact of exercise on physiological stress among patients with coronary heart disease, this study focused on CAT, GSH‐Px, and ox‐LDL as key indicators of the body's oxidation and antioxidant balance. The results revealed that both exercise programs significantly improved CAT and GSH‐Px levels. Following a 16‐week mixed Tai Chi cardiac exercise rehabilitation programme, changes in CPSS scores were negatively correlated with changes in the activity of the antioxidant enzyme GSH‐Px (*r* = −0.585, *p* < 0.05) and positively correlated with changes in ox‐LDL (*r* = 0.569, *p* < 0.05) in the experimental group, whereas no such correlations were observed in the control group.

Compared with regular aerobic exercise, mind‐body exercise offers advantages in regulating psychological states such as stress, anxiety, and depression, as well as in relieving pain. A previous review also found that Tai chi involves continuous slow movements with small‐to‐large expressions of motion, unilateral‐to‐bilateral shifts of body weight, and rotation of the trunk, head, and extremities, combined with deep diaphragmatic breathing and relaxation (Yin et al. 2023). Importantly, Tai Chi offers significant advantages for enhancing physical strength and supporting individuals in sustaining high energy levels, among other benefits (Ma et al. 2023). This finding was in agreement with the finding which is reported by You et al., motor function and sleep quality significantly improved after a 3‐month Tai Chi intervention (You et al. 2025). Additionally, Tai Chi practice enhanced muscular fitness, tactile sensation, potentially enhancing flexibility and static postural control in older adults (Bai et al. 2023; Zhang et al. 2024). What underlies these positive effects?

First, the Tai Chi rehabilitation programme focuses on specific practice points and action requirements, emphasising abdominal breathing and mental concentration. Mind‐body exercise positively impacts stress perception by reducing both physical and mental stress through techniques such as breathing exercises, yoga, and meditation (Horiuchi et al. 2022). These exercises involve specific movement patterns, body postures, deep diaphragmatic breathing, meditative mental states, and relaxed mental states (Li et al. 2024). Second, the practice of Tai Chi can regulate physiological responses and reduce the levels of stress hormones, such as cortisol, in the body (Guyon 2024). A previous study revealed that individuals who regularly practice Tai Chi in stressful situations experience a significant reduction in their body's stress response, leading to an improved psychological state (Zhang et al. 2011). This physiological change enables individuals to better cope with stress in their daily lives.

Finally, studies have demonstrated that Tai Chi has a significant positive effect on mental health. Practicing Tai Chi enables individuals to reduce their levels of anxiety, depression, and stress effectively. Research indicates that Tai Chi not only improves mental health but also enhances mental resilience, allowing individuals to better cope with and adapt to stressful situations (Wang et al. 2010; Wang et al. 2013).

In summary, the findings of the current study suggest that practicing Tai Chi enhance overall bodily functions through approaches such as balancing the body, controlling the breath, and calming the mind. The utilization of deep and prolonged breathing techniques can increase lung and chest elasticity, increase the contractile strength of the heart (Xu et al. 2021; Castro et al. 2022), and improve the efficiency and coordination of respiration (Maris et al. 2024). Exercises derived from traditional Chinese medicine are adaptable to various environments and are relatively easy to master, thereby facilitating consistent practice. These forms of exercise play a vital role in addressing cardiovascular risk factors, enhancing heart performance, and ultimately improving overall quality of life, serving as a valuable complement to Western medical treatments.

## 5 Conclusions

A 12‐week hybrid Tai Chi cardiac rehabilitation programme has been shown to help regulate psychological stress and emotions and reduce cellular stress levels in patients with coronary heart disease. This programme may alleviate patients' perception of psychological stress by reducing the damage caused by lipid metabolism stress products and increasing antioxidant enzyme activity. The promotion of comprehensive Tai Chi exercise programs to offer a variety of exercise rehabilitation options for elderly patients with coronary heart disease in the community is recommended.

## Conflicts of Interest

The authors declare that they have no known competing financial interests or personal relationships that could have appeared to influence the work reported in this paper.

## Data Availability

The datasets used and/or analysed during the current study are available from the corresponding author upon reasonable request.
